# Pathophysiology of exercise intolerance in chronic diseases: the role of diminished cardiac performance in mitochondrial and heart failure patients

**DOI:** 10.1136/openhrt-2017-000632

**Published:** 2017-07-28

**Authors:** Jodi McCoy, Matthew Bates, Christopher Eggett, Mario Siervo, Sophie Cassidy, Jane Newman, Sarah A Moore, Grainne Gorman, Michael I Trenell, Lazar Velicki, Petar M Seferovic, John G F Cleland, Guy A MacGowan, Doug M Turnbull, Djordje G Jakovljevic

**Affiliations:** 1 Institute of Cellular Medicine, Medical School, Newcastle University, Newcastle upon Tyne, UK; 2 Department of Cardiothoracic, The James Cook University Hospital, Middleborough, UK; 3 Institute of Neurosciences, Newcastle University, Newcastle upon Tyne, UK; 4 Wellcome Trust Centre for Mitochondrial Research, Newcastle University, Newcastle uponTyne, UK; 5 Research Councils UK Centre for Ageing and Vitality, Newcastle University, Newcastle upon Tyne, UK; 6 Department of Cardiovascular Surgery and Faculty of Medicine, Institute of Cardiovascular Diseases Sremska Kamenica, Novi Sad, Serbia; 7 Department of Cardiology, Clinical Centre Serbia, University of Belgrade, Serbia, UK; 8 Department of Cardiology, Imperial College Royal Brompton and Harefield Trust London, London, UK; 9 Department of Cardiology, Freeman Hospital and Institute of Genetic Medicine, Newcastle University, Newcastle upon Tyne, UK; 10 Clinical Research Facility, Royal Victoria Infirmary, Newcastle upon Tyne, UK

**Keywords:** exercise limitations, heart failure, cardiac function, oxygen consumption

## Abstract

**Objective:**

Exercise intolerance is a clinical hallmark of chronic conditions. The present study determined pathophysiological mechanisms of exercise intolerance in cardiovascular, neuromuscular, and metabolic disorders.

**Methods:**

In a prospective cross-sectional observational study 152 patients (heart failure reduced ejection fraction, n=32; stroke, n=34; mitochondrial disease, n=28; type two diabetes, n=28; and healthy controls, n=30) performed cardiopulmonary exercise testing with metabolic and haemodynamic measurements. Peak exercise O_2_ consumption and cardiac power output were measures of exercise tolerance and cardiac performance.

**Results:**

Exercise tolerance was significantly diminished in patients compared with controls (ie, by 45% stroke, 39% mitochondria disease, and 33% diabetes and heart failure, p<0.05). Cardiac performance was only significantly reduced in heart failure (due to reduced heart rate, stroke volume, and blood pressure) and mitochondrial patients (due reduced stroke volume) compared with controls (ie, by 53% and 26%, p<0.05). Ability of skeletal muscles to extract oxygen (ie, arterial-venous O_2_ difference) was diminished in mitochondrial, stroke, and diabetes patients (by 24%, 22%, and 18%, p<0.05), but increased by 21% in heart failure (p<0.05) compared with controls. Cardiac output explained 65% and 51% of the variance in peak O_2_ consumption (p<0.01) in heart failure and mitochondrial patients, whereas arterial-venous O_2_ difference explained 69% (p<0.01) of variance in peak O_2_ consumption in diabetes, and 65% and 48% in stroke and mitochondrial patients (p<0.01).

**Conclusions:**

Different mechanisms explain exercise intolerance in patients with heart failure, mitochondrial dysfunction, stroke and diabetes. Their better understanding may improve management of patients, their stress tolerance and quality of life.

**Key questions**

**What is already known about this subject?**

- Exercise intolerance is a clinical hallmark of chronic conditions and strong predictor of morbidity and mortality.

**What does this study add?**

- The study highlights that pathophysiological mechanisms of exercise intolerance differ among patients groups.

**How might this impact on clinical practice?**

- Clinical care teams may improve management of patients, their stress tolerance and quality of life by prescribing appropriate interventions specifically targeting the underlying cause of exercise intolerance that is, heart function and / or ability of skeletal muscles to extract oxygen.

## Introduction

Exercise intolerance is a clinical hallmark of chronic diseases associated with increased morbidity and mortality, and reduced quality of life for patients.^1–7^ Healthy individuals with diminished exercise tolerance demonstrate higher rates of mortality, and increased incidence of heart failure and coronary artery disease.^1 2 8^ Similarly, patients with type two diabetes, stroke, heart failure, pulmonary, and neuromuscular disorders presented with lower level of exercise tolerance show increased rate of disease progression and mortality.^3–7^ It is therefore not surprising that exercise intolerance has been an important therapeutic target in chronic conditions using both pharmacological and physiological interventions.^9–13^

Exercise intolerance is a complex clinical syndrome represented with reduced oxygen (O_2_) consumption during physiological stimulation.^14^ Aetiology of exercise intolerance can be explained by diminished capacity of the cardiovascular system to supply oxygen (heart function and cardiac output), and inability of the skeletal muscles to utilise delivered oxygen (mitochondrial function), or both.^14^ In healthy individuals exercise capacity seems to be limited by the ability of the cardiovascular system to deliver oxygen to the exercising muscles.^15^ In patients with chronic conditions, that is,. diabetes mellitus, cardiovascular, pulmonary, and neuromuscular disorders, the pathophysiology of exercise intolerance is not well understood with evidence supporting mechanisms associated with peripheral and/or central (cardiac) limitations to exercise.^16–22^ Although aetiology of exercise intolerance in heart failure has been studied more extensively than any other chronic condition, the evidence so far has been equivocal.^23–29^

Better understanding of mechanisms of exercise intolerance is important as it may lead to improved patient management and quality of life. Therefore, the present study was designed to determine underlying pathophysiological mechanisms of exercise intolerance in patients with different chronic conditions. We tested the hypothesis that diminished cardiac performance is the major cause of exercise intolerance in cardiovascular, neuromuscular, and metabolic disorders.

## Methods

### Study design

Prospective, single-centre, cross-sectional, observational study evaluated mechanisms of exercise intolerance in patients with cardiovascular, neuromuscular, and metabolic disorders. Data were collected between September 2012 and January 2016, and analysed between February and June 2016. The study was approved by the Research Ethics Committee North East of England - Tyne and Wear South.

### Participants

One hundred and fifty two patients were recruited to participate in this study. Study participants included: 1) 32 patients with stable chronic heart failure with reduced left ventricular ejection fraction (LVEF, 32%±9%; New York Heart Association functional class II and III), 2) 34 patients with a history of ischaemic stroke >6 months prior to the study (National Institutes of Health Stroke Scale average score of 3, ranging from 0 to 8) with no concomitant history of heart failure. All stroke patients were able to mobilise independently with/without a stick for 6 min; 3) 28 patients with mitochondrial disease related to either the m.3243A >G or m.8344A>G mutation with clinical stability for >6 months and no history of coronary artery disease or heart failure. Disease burden was mild or moderate in all patients (14 patients had MIDD, 12 had myopathic phenotype and two patients had MELAS). 4) 28 patients with well-controlled type two diabetes for >6 months with no history of coronary artery disease or heart failure, and average HbA1c of 7.0%±0.8%; and 5) 30 healthy individuals with no history of disease or contraindications to exercise testing. Comprehensive screening was completed in all patients including a detailed medical history, physical examination, blood pressure, ECG, and exercise stress testing. Participants were excluded from the study if they had an absolute contraindication to cardiopulmonary exercise stress testing previously suggested.^30^ All participants provided informed written consent according to the Declaration of Helsinki.

### Procedures

All participants underwent graded cardiopulmonary exercise testing using an electromagnetically controlled semi-recumbent bicycle ergometer (Corival; Lode, Groningen, The Netherlands) with non-invasive gas-exchange (Metalyzer 3B, Cortex, Leipzig, Germany) and cardiac output with the bioreactance method (NICOM, Cheetah Medical, Delaware).^31 32^ The signal processing unit of the NICOM determines the relative phase shift (Δα) between input signals relative to the output signal. The Δα is in response to any changes in blood flow that pass through the aorta. Cardiac output is then derived by Cardiac output=(C×VET×Δα dt_max_)×HR, where C is the constant of proportionality and VET is ventricular ejection fraction time.^31 32^ The value of C has been previously validated to account for patient age, gender, height and weight. Stroke volume can then be calculated from cardiac output and heart rate.

Online expired gas was measured to determine peak O_2_ consumption, along with electrocardiography (standard 12-lead configuration appropriate for exercise testing), and non-invasive blood pressure monitoring (brachial artery cuff sphygmomanometery). Cardiovascular and metabolic measurements were monitored and analysed during the 5 min rest period and throughout exercise protocol. All participants performed exercise protocol until they reached volitional exhaustion, or were unable to continue cycling at the required cadence of between 60–70 revolutions per minute. Peak exercise was defined as the absence of any rise in oxygen consumption when exercise intensity was increased, inability of the patient to continue to pedal at the required cadence, or achieved respiratory exchange ratio >1.1. Peak O_2_ consumption was defined as the average oxygen uptake in the last 30 s of exercise. Cardiac power output (watts), as the measure of overall cardiac function and performance,^33^ was calculated using the following equation: CPO = (Q_T_ x MAP) x 2.22×10^–3^, where Q_T_ is cardiac output, MAP is the mean arterial pressure, and 2.22×10^–3^ is the conversion factor.^34^ Arteriovenous oxygen difference (a-vO_2_), was calculated as the ratio between O_2_ consumption and cardiac output, represents the ability of the skeletal muscles to extract delivered O_2_.^14^

### Statistical analyses

Statistical analysis was carried out using SPSS version 21 (SPSS, Inc., Chicago, Illinois). Prior to analysis, all data were screened for univariate outliers using Z-distribution cut-off scores, and multivariate outliers were detected via the Mahalanobis distance test. A Kolmogorov-Smirnov test was used to assess the normality of distribution of the data. To test differences in measured variables between the patients groups, a one-way analysis of variance was used. To identify the groups that differed significantly from one another, a post-hoc Tukey's test was performed. The relationship between exercise tolerance and its determinants was assessed using Pearson's product moment coefficient of correlation (r). The meaningfulness of the coefficient of correlation was evaluated by calculating the coefficient of determination (R.^2^ Statistical significance was indicated if p<0.05. All data are presented as means (SD, SD) unless otherwise indicated.

## Results

Study participants demographic details are presented in table 1. Patients with mitochondrial disease had a significantly lower age (p<0.01) compared with other groups.

**Table 1:** Participants’ Demographic and Clinical Characteristics

	Healthy Controls (n=30)	Diabetes (n=28)	Stroke (n=34)	Mitochondrial Disease (n=28)	Heart Failure (n=32)
Age, y	55 (12)*	60 (9)†	62 (7)‡	48 (9)§	62 (11)
Male, No. (%)	23 (75)	22 (80)	27 (80)	20 (70)	25 (77)
Weight, Kg	76.8 (12.7)¶	91.1 (12.2)†	83.0 (14.3)‡	66.1 (14.9)	79.6 (17.3)
Height, Cm	170 (10)	171 (8)	175 (7)	171 (9)	172 (10)
Body Mass Index, kg/m^2^	27 (4)¶	31 (5)**,††, †	27 (4)	26 (5)‡	26.6 (4.0)
Body Surface Area, m^2^	1.9 (0.1)	2.0 (0.3)	2.0 (0.2)	1.8 (0.1)†	1.9 (0.2)
History of Coronary Artery Disease, No. (%)	–	–	7 (21)	–	15 (48)
History of Hypertension, No. (%)	–	11 (40)	27 (80)	7 (25)	23 (71)
History of Hyperlipidemia, No. (%)	–	17 (60)	19 (55)	10 (36)	17 (47)
History of Diabetes	–	28 (100)	11 (33)	15 (55)	8 (24)
Receiving Metformin, No. (%)	–	20 (70)	–	8 (30)	6 (18)
Receiving Insulin, N. (%)	–	–	–	–	2 (6)
Receiving ACE or ARB, No. (%)	–	11 (40)	27 (80)	13 (45)	30 (94)
Receiving β-blocker, No. (%)	–	–	9 (25)	3 (10)	32 (100)
Calcium channel blocker, No. (%)	–	–	5 (15)	3 (10)	8 (24)
Receiving statin	–	17 (60)	19 (55)	11 (36)	30 (94)
Receiving Antiarrhythmic, No. (%)	–	–	9 (25)	4 (15)	15 (47)

Significant differences between groups (significanc e p<0.05, data ext rapolated from on e-way ANOVA an d Tukey post-hoc test):

*Mitochondrial Disease vs Healthy

†Mitochondrial Disease vs Diabetes

‡Stroke vs Mitochondrial Disease

§Heart Failure vs Mitochondrial Disease

¶Diabetes vs Healthy

**Heart Failure vs Diabetes

††Stroke vs Diabetes

ACE, angiotensin-converting enzyme; ARB, angiotensin receptor blocker

### Measurements at rest

**Figure 1:**
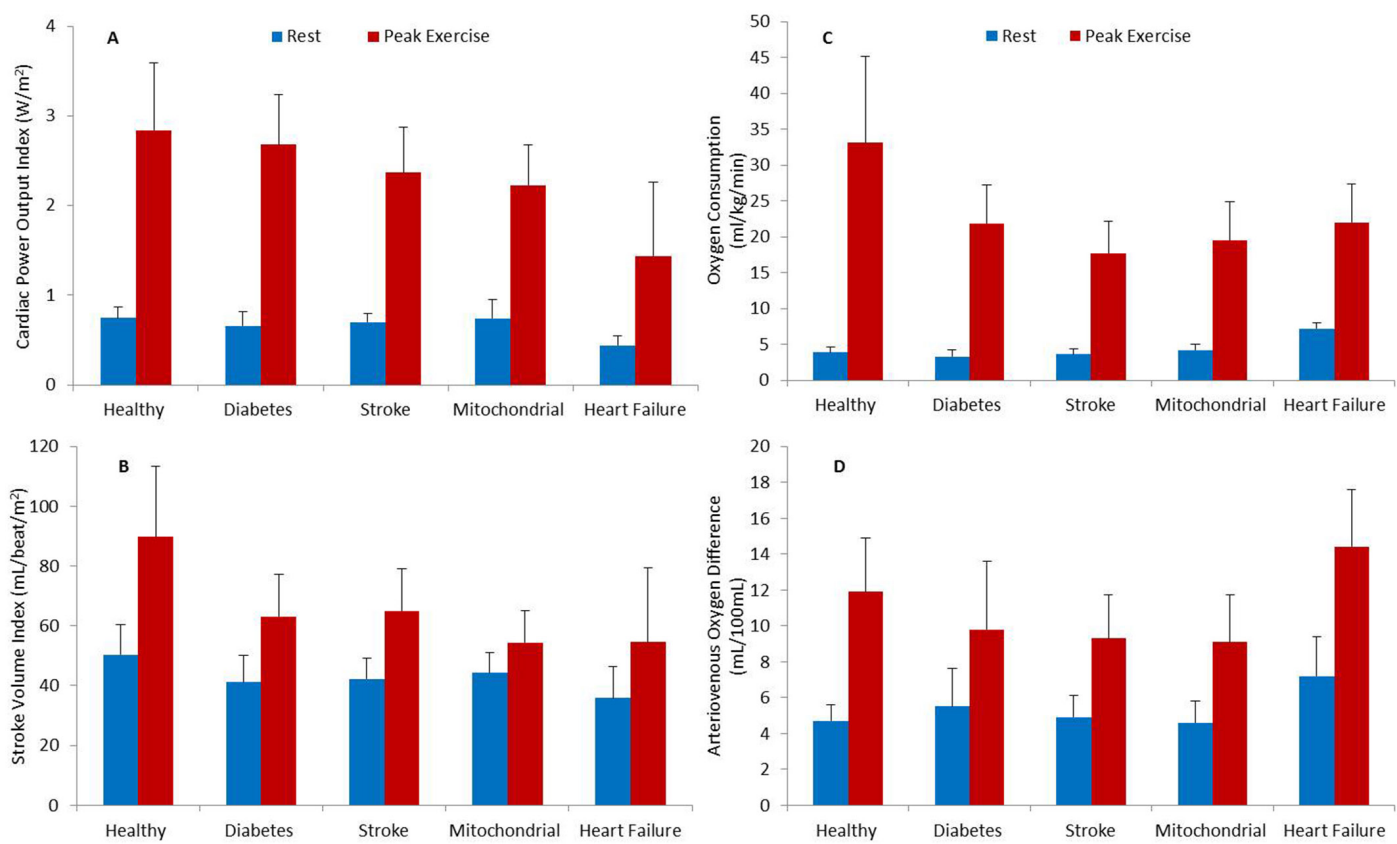
Central haemodynamic and functional capacity variables measured at rest and peak exercise that is, Cardiac power output index (**A**), Stroke volume index (**B**), Oxygen consumption (**C**), Arteriovenous oxygen difference (**D**) in different patients groups.

The heart failure patients demonstrated significantly lower values of cardiac variables including cardiac power output, blood pressure, and cardiac output compared with other groups (p<0.05; table 2). Resting heart rate was highest in mitochondrial disease, which was significantly higher than in heart failure and healthy participants (27% and 15% respectively, p<0.05). Cardiac power output index and stroke volume index were significantly lower in heart failure patients compared with healthy controls (p<0.05, figure 1A-B). Oxygen consumption was similar between the groups, with mitochondrial disease only demonstrating higher values than diabetes (p<0.05, table 2, figure 1C). Arterial-venous O_2_ difference was also similar between the groups, except heart failure demonstrating significantly higher values (table 2, figure 1D).

**Table 2:** Resting and Peak Exercise Cardiovascular and Metabolic Variables. Data presented as mean (SD).

	Healthy Controls (n=30)	Diabetes (n=28)	Stroke (n=34)	Mitochondrial Disease (n=28)		Heart Failure (n=32)
*Resting metabolic and cardiac variables*						
Oxygen consumption, mL/kg/min	3.9 (0.8)	3.3 (1.0)*	3.6 (0.8)	4.2 (0.8)		4.0 (0.8)
Heart rate, bpm	69 (8)†	76 (10)‡	74 (10)§	79 (10)¶		62 (10)
Mean blood pressure, mmHg	101 (10)	106 (11)‡	102 (11)	108 (16)¶		92 (9)
Cardiac index, L/min/m^2^	3.5 (0.6)**	3.1 (0.4)‡	3.2 (0.5)§	3.4 (0.4)¶		2.2 (0.2)
Cardiac power output, Watts	1.41 (0.29)**	1.33 (0.28)‡	1.39 (0.27)§	1.48 (0.41) ¶		0.86 (0.23)
Stroke Volume, mL/beat	95.1 (22.9)**, †	83.0 (15.3)	83.7 (13.9)	77.8 (13.2)		68.4 (17.1)
Peripheral vascular resistance, dyne/s/cm^2^	Ddsd 1215 (252) **, †	1368 (308)	1275 (296)	1416 (351)		1795 (382)
Arteriovenous oxygen difference, mL/100 mL	4.7 (0.9)**	5.5 (2.1)‡	4.9 (1.2)§	4.6 (1.2) ¶		7.2 (2.2)
*Peak exercise metabolic and cardiac variables*						
Oxygen consumption, mL/kg/min	33.1 (12.1)**, ††, †, ‡‡	21.9 (5.3)	17.7 (4.5)	19.5 (5.4)		22.0 (5.3)
Heart rate, bpm	157 (24)**, *	153 (14)‡, §§	127 (26)§	150 (24)¶, **		117 (25)
Oxygen pulse, mL/beat	16.2 (4.4)**, *, †, ††	13.0 (4.1)‡, *	11.6 (3.9)	8.6 (3.2)¶		12.2 (4.5)
Mean blood pressure, mmHg	130 (11)**	140 (16)‡, *	133 (9)§	125 (13)¶		105 (15)
Cardiac index, L/min/m^2^	9.9 (2.4)**, †	8.6 (1.9)‡	8.0 (1.2)§	8.4 (1.6)		6.1 (1.1)
Cardiac power output, Watts	5.41 (1.70)**, †	5.37 (1.11)‡, *	4.71 (1.14)§	3.98 (1.11)		2.56 (1.53)
Stroke Volume, mL/beat	125.7 (36.9)**, †	126.8 (29.1)§§	128.9 (30.1)	96.5 (24.7)		102.9 (43.5)
Peripheral vascular resistance, dyne/s/cm^2^	553 (128)^**^	651 (192)	665 (177)	662 (204)		724 (298)
Arteriovenous oxygen difference, mL/100 mL	11.9 (3.0)**, *, †	9.8 (3.8)‡	9.3 (2.4)§	9.1 (2.6)^¶^		14.4 (4.2)
Ventilatory efficiency slope	27.2 (6.4)**, *, †	29.8 (8.1)‡, *, §§	32.4 (7.4)	35.7 (8.3)		36.7 (7.8)

Significant differences between the groups (significance p<0.05, data extrapolated from one-way ANOVA and Tukey post-hoc test):

*Mitochondrial Disease vs Diabetes

†Mitochondrial Disease vs Healthy

‡Heart Failure vs Diabetes

§Heart failure vs Stroke

¶Heart Failure vs Mitochondrial Disease

**Heart Failure vs Healthy

††Stroke vs Healthy

‡‡Diabetes vs Healthy

§§Stoke vs Diabetes

¶¶Stoke vs Mitochondrial Disease

### Exercise tolerance and its determinants

All participants demonstrated a significant effort during cardiopulmonary exercise testing reflected with peak exercise respiratory exchange ratio >1.10 (healthy, 1.16±0.10; diabetes, 1.14±0.11; stroke, 1.10±0.06; mitochondria, 1.13±0.09; and heart failure, 1.12±0.08). Forty-two % stroke patients terminated exercise test before reaching respiratory exchange ratio >1.10. Similarly, in 22% and 26% of mitochondrial and heart failure patients were not able to continue to pedal at the required cadence before reaching respiratory exchange ratio of >1.10.

Anaerobic threshold, expressed as a percentage of achieved peak oxygen consumption, was achieved in all patients, and was significantly reduced in patients compared with controls (healthy, 62±12; diabetes, 52±13; stroke, 46±8; mitochondria, 44±14; and heart failure, 48±9, p<0.05).

Peak exercise O_2_ consumption was significantly diminished in patients compared with controls that is, by 33% in diabetes and heart failure, 39% in mitochondrial disease, and 45% in stroke (p<0.05, table 2, figure 1C). Peak exercise cardiac output was only significantly reduced in heart failure and mitochondrial patients compared with controls i.e. by 59% and 30% (p<0.05), respectively as was cardiac power output by 53% and 26% (p<0.05, table 2, figure 1A). Cardiac performance was diminished due to reduced heart rate (25%), stroke volume (18%), and mean arterial blood pressure (19%) in heart failure (p<0.05), and stroke volume (23%) in mitochondrial patients (p<0.05) compared with healthy controls. Arterial-venous O_2_ difference was significantly reduced in stroke, mitochondrial, and diabetes patients compared with healthy controls that is, by 22%, 24%, and 18% (p<0.05), but significantly increased in heart failure by 21% (p<0.05, table 2, figure 1D).

### Relationship between exercise tolerance, cardiac performance and arterial-venous O_2_ difference

When data from all study participants were combined, there was a significant positive moderate relationship between peak exercise O_2_ consumption and cardiac output (r=0.59, p<0.01), cardiac power output (0.52, p<0.01), and arterial-venous O_2_ difference (r=0.50, p<0.01). Subgroup analysis revealed that exercise tolerance was highly dependent on cardiac performance in heart failure, with cardiac output explaining 65% of the variance in peak O_2_ consumption, respectively (r=0.81, p<0.01, table 3). Furthermore, ability of skeletal muscles to extract O_2_ was the major determinant of exercise tolerance in stroke and diabetes, with arterial-venous O_2_ difference explaining 65% (r=0.81, p<0.01) and 69% (r=0.83, p<0.01) of the variance in peak oxygen consumption respectively. In healthy controls and mitochondrial patients, exercise tolerance was significantly influenced by both, central and peripheral factors such that cardiac output explains 31% and 51% (p<0.01), whereas arterial-venous O_2_ difference 44% and 48% (p<0.01) of the overall variance in peak O_2_ consumption (table 3).

**Table 3:** Relationship between peak exercise oxygen consumption and cardiac output and arteriovenous oxygen difference

	O_2_ consumption ~ Cardiac output (L/min)			O_2_ consumption ~ Arterial-venous O_2_ different (mL)		
	*r*	*R*^2^	p	*r*	*R*^2^	p
Healthy	0.556	0.309	0.002	0.670	0.449	0.001
Diabetes	0.108	0.011	0.690	0.832	0.692	0.000
Stroke	0.511	0.261	0.021	0.807	0.651	0.000
Mitochondrial Disease	0.714	0.509	0.000	0.695	0.483	0.000
Heart Failure	0.808	0.652	0.000	0.160	0.026	0.555

## Discussion

The main finding of the present study suggests that pathophysiological mechanisms underlying exercise intolerance differ between the clinical groups. The major limitation to exercise in heart failure is diminished cardiac performance, whereas in stroke and diabetes the major contributor to exercise intolerance is reduced ability of skeletal muscles to extract O_2_. In mitochondrial patients exercise tolerance was significantly influenced by both, central and peripheral factors. These findings may have important clinical implications that is, that therapeutic pharmacological and physiological interventions targeting exercise intolerance need to be specific and tailored towards clinical presentation of an individual patient and in the line with aetiology of functional impairment. Results from the present study add novelty to the existing literature by providing for the first time direct comparison between mechanisms of exercise intolerance among patients with different underlying chronic conditions. Exercise intolerance in a clinical hallmark of many chronic diseases and better understanding of its pathology may lead to better patient management and clinical outcomes.

As expected exercise tolerance was significantly diminished in patients compared with healthy subjects. However, peak O_2_ consumption interestingly was not significantly different among the patient groups and was in the range of ~10% from each other. Heart failure with reduced ejection fraction and diabetes patients demonstrated similar level of exercise tolerance which is by ~30% lower than in healthy controls. However, the mechanisms of exercise intolerance are clearly distinct with cardiac performance (ie, peak exercise cardiac output and cardiac power output) being reduced in heart failure by ~40% and~50% compared with healthy controls. This finding is supported by previous investigations in heart failure due to left ventricular systolic dysfunction suggesting that the major determinant of exercise tolerance is impaired cardiac function, whereas a significant arterial-venous O_2_ difference reserve suggests skeletal muscle per se contributes minimally to limiting exercise capacity.^27–29^

Contrary to heart failure, patients with diabetes demonstrate cardiac performance which is only ~10% lower than healthy controls. This may be a surprising finding considering susceptibility of patients with diabetes to cardiac dysfunction and heart failure even in the absence of coronary artery disease, hypertension, and valvular disease.^35 36^ However, in agreement with our findings, previous studies also reported that peripheral mechanisms and skeletal muscles ability to extract delivered O_2_ was the main determinant of the O_2_ uptake during exercise in patients with type two diabetes.^16 22^ As previously suggested type two diabetic skeletal muscle demonstrates a transient imbalance of muscle oxygen delivery relative to oxygen uptake after onset of exercise, suggesting a slowed microvascular blood flow increase in type two diabetic muscle.^16^ Impaired vasodilatation due to vascular dysfunction in type two diabetes during exercise may contribute to this observation.^16^

Similarly as in diabetes, the major mechanisms of exercise intolerance in stroke patients was not cardiac performance but rather significantly reduced ability of skeletal muscles to extract O_2_, explaining remarkable ~65% of the variance in measured O_2_ consumption. A limited number of studies have reported pathophysiology of exercise intolerance in stroke. While it was initially argued that cardiac performance that is, cardiac output to be reduced by ~1/3 in stroke patients,^18^ later study reported that cardiac function response to exercise is preserved,^17^ confirming the finding of the present study that exercise capacity is predominantly effected by skeletal muscle dysfunction. People who suffer from stroke are often left with residual physical impairments limiting physical function. Unique structural and metabolic abnormalities have been demonstrated in hemiparetic muscle post-stroke which may be a direct result of stroke or caused by an indirect reduction in physical activity levels due to impairment further exacerbating reduction in skeletal muscle mass.^37^ The neurological involvement of the individual may also play a key role in determining exercise intolerance, for example, spasticity of the lower extremities, or poor motor coordination.^5^

Mitochondrial disease patients, despite being the youngest group, demonstrated functional capacity that was ~12% lower than that of heart failure. When compared with healthy controls, mitochondrial patients showed significantly diminished both, cardiac and peripheral muscle performance by ~25%, with the lowest arterial-venous O_2_ difference among the patient groups. It is interesting to note that, in contrast with other clinical groups, reduced exercise tolerance in patients with mitochondrial disease was equally affected by both, central and peripheral factors with cardiac output and arterial-venous O_2_ difference explaining 31% and 51% of the variance in O_2_ consumption. Mitochondrial disease patients do not seem to have the compensatory mechanism seen in heart failure, possibly due to an increased defective mitochondrial content in the muscle and oxidative phosphorylation, preventing efficient oxygen uptake into the working muscles.^38^ Mitochondrial patients attempted to overcome defective arterial-venous O_2_ difference by increasing the heart rate to support peak oxygen consumption. However, overall cardiac reserve (difference between peak exercise and resting heart rate) was diminished because resting heart rate was increased. Additionally, reduced chronotropic competence is featured with reduced ability to increase stroke volume. Our findings are supported by previous studies which also highlighted the inability of mitochondrial disease patients to exceed 10 mL arterial-venous O_2_ difference due to impaired respiratory chain function, reducing the capacity of the skeletal muscles to extract delivered oxygen.^39^ This confirms the complex nature of this systemic disorder effecting both the heart and skeletal muscle.^19^

In the present study the following limitations should be considered. First, the overall sample size in each of the group was moderate and non-invasive bioreactance method was used to assess central haemodynamics. Second, the mean age of the mitochondrial patients was significantly less than the other groups. Results reveal however that most of the physiological variables were in fact lower than reported in other groups despite the younger age, and in the line with clinical presentation of mitochondrial disease patients. Thirdly, the disease associated concomitant medication was different among the groups, and particularly the use of beta-adrenergic receptor blockers which has known effect to reduce cardiac performance. Patients were however instructed not to withdraw medication prior assessment. Lastly, the study was not designed to evaluate other factors (in addition to cardiac output and arterial-venous oxygen difference) that may contribute to impaired oxygen consumption during exercise (including muscle biochemistry, microcirculation, pulmonary discussion capacity, and oxygen carrying capacity), as previously suggested.^14 15^

## Conclusion

The present study suggest that pathophysiology of exercise intolerance differ among patients group. Diminished cardiac performance plays dominant role in exercise tolerance in patients with heart failure with reduced ejection fraction, whereas arterial-venous O_2_ difference is the major contributor in patients with diabetes and stroke. Exercise capacity in patients with mitochondrial disorders is equally affected by both central haemodynamic and peripheral factors. These findings may have important clinical implications because better understanding of the pathophysiology of exercise intolerance in chronic diseases may improve management of the patients, their stress tolerance and quality of life.

## References

[1] Blair SN, Kohl HW, Paffenbarger RS, et al Physical fitness and all-cause mortality. A prospective study of healthy men and women. JAMA 1989;262:2395–401. 2795824 10.1001/jama.262.17.2395

[2] Berry JD, Pandey A, Gao A, et al Physical fitness and risk for heart failure and coronary artery disease. Circ Heart Fail 2013;6:627–34. 10.1161/CIRCHEARTFAILURE.112.000054 23677924 PMC5152944

[3] Mancini DM, Eisen H, Kussmaul W, et al Value of peak exercise oxygen consumption for optimal timing of cardiac transplantation in ambulatory patients with heart failure. Circulation 1991;83:778–86. 10.1161/01.CIR.83.3.778 1999029

[4] Wei M, Gibbons LW, Kampert JB, et al Low cardiorespiratory fitness and physical inactivity as predictors of mortality in men with type 2 diabetes. Ann Intern Med 2000;132:605–11. 10.7326/0003-4819-132-8-200004180-00002 10766678

[5] Gordon NF, Gulanick M, Costa F, et al Physical activity and exercise recommendations for stroke survivors. Circulation 2004;109:2031–41. 15117863 10.1161/01.CIR.0000126280.65777.A4

[6] Burns JM, Cronk BB, Anderson HS, et al Cardiorespiratory fitness and brain atrophy in early alzheimer disease. Neurology 2008;71:210–6. 10.1212/01.wnl.0000317094.86209.cb 18625967 PMC2657657

[7] Wensel R, Opitz CF, Anker SD, et al Assessment of survival in patients with primary pulmonary hypertension: importance of cardiopulmonary exercise testing. Circulation 2002;106:319–24. 10.1161/01.CIR.0000022687.18568.2A 12119247

[8] Kodama S, Saito K, Tanaka S, et al Cardiorespiratory fitness as a quantitative predictor of all-cause mortality and cardiovascular events in healthy men and women: a meta-analysis. JAMA 2009;301:2024–35. 10.1001/jama.2009.681 19454641

[9] Wenger NK Lifestyle interventions to improve exercise tolerance in obese older patients with heart failure and preserved ejection fraction. JAMA 2016;315:31–3. 10.1001/jama.2015.17347 26746454

[10] Edelmann F, Wachter R, Schmidt AG, et al Effect of spironolactone on diastolic function and exercise capacity in patients with heart failure with preserved ejection fraction: the Aldo-DHF randomized controlled trial. JAMA 2013;309:781–91. 10.1001/jama.2013.905 23443441

[11] Cohn JN Vasodilators in heart failure. conclusions from V-HeFT II and rationale for V-HeFT III. Drugs 1994;47(Suppl 4):47–57. 7523062 10.2165/00003495-199400474-00008

[12] Gullestad L, Manhenke C, Aarsland T, et al Effect of metoprolol CR/XL on exercise tolerance in chronic heart failure - a substudy to the MERIT-HF trial. Eur J Heart Fail 2001;3:463–8. 11511433 10.1016/s1388-9842(01)00146-5

[13] Piña IL, Apstein CS, Balady GJ, et al Exercise and heart failure: a statement from the American Heart Association Committee on exercise, rehabilitation, and prevention. Circulation 2003;107:1210–25. 10.1161/01.CIR.0000055013.92097.40 12615804

[14] Jones NL, Killian KJ Exercise limitation in health and disease. N Engl J Med 2000;343:632–41. 10.1056/NEJM200008313430907 10965011

[15] Bassett DR, Howley ET Limiting factors for maximum oxygen uptake and determinants of endurance performance. Med Sci Sports Exerc 2000;32:70–84. 10.1097/00005768-200001000-00012 10647532

[16] Bauer TA, Reusch JE, Levi M, et al Skeletal muscle deoxygenation after the onset of moderate exercise suggests slowed microvascular blood flow kinetics in type 2 diabetes. Diabetes Care 2007;30:2880–5. 10.2337/dc07-0843 17675540

[17] Jakovljevic DG, Moore SA, Tan LB, et al Discrepancy between cardiac and physical functional reserves in stroke. Stroke 2012;43:1422–5. 10.1161/STROKEAHA.111.649434 22363066

[18] Tomczak CR, Jelani A, Haennel RG, et al Cardiac reserve and pulmonary gas exchange kinetics in patients with stroke. Stroke 2008;39:3102–6. 10.1161/STROKEAHA.108.515346 18703810

[19] Bates MG, Newman JH, Jakovljevic DG, et al Defining cardiac adaptations and safety of endurance training in patients with m.3243A>G-related mitochondrial disease. Int J Cardiol 2013;168:3599–608. 10.1016/j.ijcard.2013.05.062 23742928 PMC3819621

[20] Sun XG, Hansen JE, Oudiz RJ, et al Exercise pathophysiology in patients with primary pulmonary hypertension. Circulation 2001;104:429–35. 10.1161/hc2901.093198 11468205

[21] Grewal J, McCully RB, Kane GC, et al Left ventricular function and exercise capacity. JAMA 2009;301:286–94. 10.1001/jama.2008.1022 19155455 PMC2862454

[22] Baldi JC, Aoina JL, Oxenham HC, et al Reduced exercise arteriovenous O_2_ difference in type _2_ diabetes. J Appl Physiol 2003;94:1033–8. 10.1152/japplphysiol.00879.2002 12571134

[23] Clark AL, Poole-Wilson PA, Coats AJ Exercise limitation in chronic heart failure: central role of the periphery. J Am Coll Cardiol 1996;28:1092–102. 10.1016/S0735-1097(96)00323-3 8890800

[24] Maeder MT, Thompson BR, Brunner-La Rocca HP, et al Hemodynamic basis of exercise limitation in patients with heart failure and normal ejection fraction. J Am Coll Cardiol 2010;56:855–63. 10.1016/j.jacc.2010.04.040 20813283

[25] Nilsson KR, Duscha BD, Hranitzky PM, et al Chronic heart failure and exercise intolerance: the hemodynamic paradox. Curr Cardiol Rev 2008;4:92–100. 10.2174/157340308784245757 19936283 PMC2779357

[26] Ponikowski PP, Chua TP, Francis DP, et al Muscle ergoreceptor overactivity reflects deterioration in clinical status and cardiorespiratory reflex control in chronic heart failure. Circulation 2001;104:2324–30. 10.1161/hc4401.098491 11696473

[27] Esposito F, Mathieu-Costello O, Shabetai R, et al Limited maximal exercise capacity in patients with chronic heart failure: partitioning the contributors. J Am Coll Cardiol 2010;55:1945–54. 10.1016/j.jacc.2009.11.086 20430267 PMC4780578

[28] Dhakal BP, Malhotra R, Murphy RM, et al Mechanisms of exercise intolerance in heart failure with preserved ejection fraction: the role of abnormal peripheral oxygen extraction. Circ Heart Fail 2015;8:286–94. 10.1161/CIRCHEARTFAILURE.114.001825 25344549 PMC5771713

[29] Spee RF, Niemeijer VM, Wessels B, et al Characterization of exercise limitations by evaluating individual cardiac output patterns: a prospective cohort study in patients with chronic heart failure. BMC Cardiovasc Disord 2015;15:57 10.1186/s12872-015-0057-6 26100151 PMC4476170

[30] Fletcher GF, Ades PA, Kligfield P, et al Exercise standards for testing and training: a scientific statement from the american Heart Association. Circulation 2013;128:873–934. 10.1161/CIR.0b013e31829b5b44 23877260

[31] Jones TW, Houghton D, Cassidy S, et al Bioreactance is a reliable method for estimating cardiac output at rest and during exercise. Br J Anaesth 2015;115:386–91. 10.1093/bja/aeu560 25659999

[32] Jakovljevic DG, Trenell MI, MacGowan GA Bioimpedance and bioreactance methods for monitoring cardiac output. Best Pract Res Clin Anaesthesiol 2014;28:381–94. 10.1016/j.bpa.2014.09.003 25480768

[33] Jakovljevic DG, George RS, Donovan G, et al Comparison of cardiac power output and exercise performance in patients with left ventricular assist devices, explanted (recovered) patients, and those with moderate to severe heart failure. Am J Cardiol 2010;105:1780–5. 10.1016/j.amjcard.2010.01.362 20538130

[34] Williams SG, Cooke GA, Wright DJ, et al Peak exercise cardiac power output; a direct indicator of cardiac function strongly predictive of prognosis in chronic heart failure. Eur Heart J 2001;22:1496–503. 10.1053/euhj.2000.2547 11482923

[35] Aneja A, Tang WH, Bansilal S, et al Diabetic cardiomyopathy: insights into pathogenesis, diagnostic challenges, and therapeutic options. Am J Med 2008;121:748–57. 10.1016/j.amjmed.2008.03.046 18724960

[36] Cassidy S, Hallsworth K, Thoma C, et al Cardiac structure and function are altered in type 2 diabetes and non-alcoholic fatty liver disease and associate with glycemic control. Cardiovasc Diabetol 2015;14:23 10.1186/s12933-015-0187-2 25849783 PMC4330943

[37] De Deyne PG, Hafer-Macko CE, Ivey FM, et al Muscle molecular phenotype after stroke is associated with gait speed. Muscle Nerve 2004;30:209–15. 10.1002/mus.20085 15266637

[38] Mancuso M, Angelini C, Bertini E, et al Fatigue and exercise intolerance in mitochondrial diseases. Literature revision and experience of the Italian Network of mitochondrial diseases. Neuromuscul Disord 2012;22(Suppl 3):S226–S229. 10.1016/j.nmd.2012.10.012 23182644 PMC3526786

[39] Taivassalo T, Jensen TD, Kennaway N, et al The spectrum of exercise tolerance in mitochondrial myopathies: a study of 40 patients. Brain 2003;126(Pt 2):413–23. 10.1093/brain/awg028 12538407

